# How Adolescents with Diabetes Experience Social Support from Friends: Two Qualitative Studies

**DOI:** 10.1155/2014/415849

**Published:** 2014-01-08

**Authors:** Louk W. H. Peters, Laura Nawijn, Nicole M. C. van Kesteren

**Affiliations:** ^1^Department of Life Style, Netherlands Organisation for Applied Scientific Research (TNO), P.O. Box 2215, 2301 CE Leiden, The Netherlands; ^2^Academic Medical Center, Department of Psychiatry, Meibergdreef 5, 1105 AZ Amsterdam, The Netherlands

## Abstract

Self-management of diabetes is challenging, especially for adolescents who face multiple changes, including closer peer relationships. Few studies have explored how friends can provide constructive support in this effort. The present research investigated, in two qualitative studies, the perceptions of adolescents with diabetes and their friends with respect to the positive social support that friends can offer. In study 1, 28 adolescents aged 12–15 with type 1 diabetes participated in online focus groups. In study 2, 11 of these adolescents were interviewed in person together with their best friends. The data were analysed by means of content analysis. In study 1, the adolescents with diabetes identified various supportive behaviours of friends, particularly concerning emotional support: treating them normally, showing interest, having fun, providing a distraction, and taking their diabetes into account. They differed in their attitude towards support, and this influenced which behaviours they perceived as supportive. Study 2 showed that the adolescents with diabetes and their friends often had similar opinions on the desired degree of support. Fear of stigmatization and sense of autonomy withheld some adolescents with diabetes from soliciting more support. These insights can be useful in patient education aiming to promote social support.

## 1. Introduction

Type 1 diabetes is a chronic illness requiring an intensive and challenging self-management regimen. This includes daily insulin injections, frequent blood glucose tests, close monitoring of food intake, and regular exercise [1]. Compared to younger children and adults with diabetes, adolescents with diabetes have been found to show poorer self-management behaviours [2] and poorer metabolic control [3, 4]. This is associated with developments characteristic of the teenage years: adolescents are becoming more independent of their parents, taking increasing responsibility for their diabetes management, and developing closer relationships with their peers [5, 6]. With regard to the latter, adolescents' desire to be accepted by their peer group can lead to poorer diabetes self-management [7]. However, greater attention has recently been paid to the role of friends as a source of positive social support in coping with diabetes [5, 8, 9]. For instance, a review tentatively concluded that social support from friends seems to have a positive influence on the self-management behaviours of adolescents with diabetes [9].

Social support can be defined as interactions between people which meet the needs of the recipient [10]. Broadly speaking, three different types of social support can be distinguished: emotional (expressions of affection, appreciation, interest, and companionship), instrumental (practical help), and informational (advice) [10]. A key element of social support is that both the type and the amount of support offered are in line with the recipient's needs.

Although increasing attention is being paid to the potentially positive contribution of social support from friends with respect to adolescents' self-management of diabetes, the evidence remains limited and the results so far have been mixed. In two studies, no relationship was found between support and self-management in general [11, 12]; in several studies a relationship was found only with specific self-management behaviours, such as glucose testing [11] or food intake [13]; and in one study a relationship was found between behaviours by friends that were perceived to be supportive and self-management in general [14].

These studies investigated social support only from the perspective of the adolescents with diabetes. Given the mixed results to date, the present study aims to gain further insight by taking a qualitative approach and focusing on the lived experiences, needs, and perspectives of the adolescents [15, 16] and by examining the interaction between adolescents with diabetes and their friends. To our knowledge, the perspectives of friends in this interaction have not yet been investigated.

Two studies are described. Study 1 examines the opinions of adolescents with diabetes on the ways in which their friends can play a positive role in helping them to cope with diabetes. Study 2 investigates the perspectives of adolescents with diabetes and their best friends on the provision of social support with respect to diabetes. Approval was granted for both studies by the Medical Ethics Committee of the Leiden University Medical Centre.

## 2. Study 1: Online Focus Groups with Adolescents with Diabetes

### 2.1. Methods

#### 2.1.1. Participants

Adolescents (aged 12–15) with type 1 diabetes were recruited by staff of four diabetes clinics in The Netherlands during regular consultation visits. Adolescents could register by returning personal and parental consent forms and a short questionnaire on background characteristics (see Table 1) to the research organization TNO. The research team contacted the adolescents by telephone and e-mail with further information about the study. After participating, the adolescents received €15 each.

Of the 19 girls and 19 boys who registered, 16 girls and 12 boys actually participated in the study. Table 1 shows that the participants had relatively diverse background characteristics, with the exception of ethnic background. Our efforts to recruit participants from migrant populations had been unsuccessful. Two online focus groups were held with girls (with 9 and 7 participants, resp.) and three with boys (with 6, 4, and 2 participants, resp.).

#### 2.1.2. Data Collection

The online focus groups were single-sex only, given that group composition should preferably be as homogeneous as possible [17] and different processes may be at work among girls and boys. For instance, boys report less support than girls [11] and seem to keep their diabetes more separate from the outside world [18]. Each group was held on 3 to 4 consecutive afternoons, with a common log-in time between 4 and 6 p.m. (synchronous focus groups, e.g., [19]). Participants were assigned a username and password to log in on a secure internet forum. They chose their own pseudonyms and avatars and remained anonymous to one another; only the moderator could see their usernames.

The moderator followed a semistructured interview protocol covering the following themes: impact of diabetes, friends' knowledge about diabetes, supportive and nonsupportive behaviours of friends, and ideas about how support could be promoted from friends. Participants were asked to respond to protocol questions and to each other by typing their entry; the forum showed all entries of all participants.

#### 2.1.3. Data Analysis

All forum entries were exported to a file and analysed using content analysis [15–17]. The entries were then grouped into the themes indicated above by way of a cyclical process of reflection, observation, and analysis. First the entries of one focus group were coded, on the basis of which the themes were supplemented as needed with other important, recurring concepts. Then the other focus groups were coded. The data were analysed by the first author and spot checks were conducted by the other authors.

### 2.2. Results

#### 2.2.1. Impact of Diabetes

Most of the participants felt that their diabetes imposed few to no restrictions on them. Some were quite unequivocal about this, reporting that they could do everything that others could. Further, many found their diabetes to be little trouble and said they were used to it. However, most participants also identified various aspects of diabetes which they found to be tedious. The most frequently mentioned in this context were the continual demands of the self-management regimen, injections, and hypo- and hyperglycaemia.

 B3: Meh, in itself I don't find it that annoying. If I have to pick something I really don't like, it would be having hypos. Those are really annoying, especially when I'm doing something active like sport or school work. (Boy, 15 years)

Psychosocial aspects were another common theme, especially the reactions of people the adolescents with diabetes did not know well. The participants reported that certain behaviours on the part of such people, such as staring, made them feel as though they were different from others.

#### 2.2.2. Disclosure

In coping with their diabetes, the participants were confronted with choices concerning what to keep to themselves and what to reveal to others. Many reported that they did not want to stand out on account of their diabetes, especially among people not well known to them. They expressed this reluctance in various ways, such as leaving the room to administer injections or being concerned by the visibility of an insulin pump. A minority of participants did not feel this sense of reserve.

 G12: I find it annoying when I'm different and I'd rather never show it, like by giving myself an injection in the classroom. I usually leave 5th period a bit early and do it at the caretaker's office… (Girl, 15 years)

The majority of the participants who showed such reserve reported being less reserved the better they knew someone. Almost all of the participants said that their friends were aware of their diabetes, although some were not entirely certain exactly what their friends knew. Moreover, many participants indicated that their friends did not fully understand the situation and that it was quite difficult to explain.

#### 2.2.3. Attitude towards Support from Friends

The participants differed in the extent to which they involved and wanted to involve their friends in their diabetes. Broadly speaking, two types can be distinguished, hereafter referred to as type A and B for ease of communication: those who did not involve or rarely involved their friends (type A) and those who were more active in that regard and were happy with friends taking their diabetes into account and helping out at times (type B). The majority of participants fell into the type B group. It is noteworthy that among boys type A seemed to be more extreme; these boys spoke of keeping their diabetes away from their friends.

 B5: My diabetes should be as far away from me as possible when I'm with my friends, otherwise you have no life. (Boy, 14 years, social support type A)

#### 2.2.4. Perceptions of Supportive and Nonsupportive Behaviour by Friends

Various forms of emotional and instrumental support were discussed in the focus groups, the former more frequently than the latter. In addition, the participants differed as to which behaviours of friends they perceived to be supportive and nonsupportive. These perceptions seemed to be related to the participants' sex and their attitudes towards support from friends.

*Emotional Support*

*(1) Normal Treatment*. Almost all participants indicated that they wanted to be treated “normally.” The exact understanding of what being treated normally meant differed mainly between type A boys and the remaining participants. For instance, the type A boys said they felt they are treated differently when friends kept diet soft drinks for them at home, while the others had no problem with this.

 B4: I think they also treat me differently, they buy all diet products and I sometimes find that annoying. I just want to be treated like everyone else, I can take care of myself. (Boy, 13 years, social support type A)

Some participants said they were bothered by friends or acquaintances who acted as though having diabetes meant nothing, as though it went away by itself, and as though it was not bad and did not hurt to have to give oneself injections.

*(2) Interest*. Many participants reported appreciating a measure of interest in their diabetes from friends, for example, asking now and then how things were going. They also saw as an expression of interest those occasions when friends asked whether they still had to have an injection, which is actually a form of instrumental support. But if their friends asked too often or tried to tell them what to do (“as though they're the boss of me”), many participants perceived this as interfering.

 B6: I think it's nice if they occasionally ask if I'm okay. But I don't like it so much if they say if I should or shouldn't do things. (Boy, 14 years, social support type B)

*(3) Fun and Distraction*. Many participants reported that simply having fun with their friends was supportive. Some said that while doing so they enjoyed not having to think about diabetes. However, they never or rarely felt down about their diabetes, and the need to be cheered up by friends was unusual. Most participants felt that they could turn to their friends if they had a problem.

 G2: It's good to just forget all the bad things and goof around. Usually we get so crazy in the (children's) disco I forget all that fussing about with diabetes and I let loose (Girl, 12 years, social support type B).

*(4) Taking the Diabetes into Account*. The participants identified various ways in which their friends took their diabetes into account: (a) keeping them company while administering an injection or waiting until they had finished, (b) having diet products at home, (c) showing solidarity (e.g., not eating when their friend could not), (d) keeping meal times in mind, and (e) asking whether plans were okay. Boys who wanted to keep their diabetes from their friends (type A) did not consider these behaviours to be supportive; they in fact saw them in a negative light because they made them feel as though they were being treated differently. Boys who did involve their friends to some extent (type B) considered examples *a* and *b* to be supportive but saw *c* as irritating: they did not want their friends to be restricted in their activities on their account.

 B6: I don't expect friends to not eat or drink certain things because I can't at that time. They don't have to make sacrifices for me. In fact, I find it irritating if they want to do that. (Boy, 14 years, social support type B)

Examples of *d* and *e* were reported only in the female groups; *e* was mentioned particularly by girls who involved their friends (type B).

*Instrumental Support*

*(1) Offering Reminders*. This form of support was discussed in the emotional support section above, having been seen by the participants as an expression of interest by friends. Only a handful of the adolescents who occasionally forgot to administer their injection found this behaviour to be supportive in an instrumental sense. Most reported paying close attention to when they were required to administer an injection, and many saw it as interfering if friends reminded them of this too often or in too directive a manner. This interference appeared to be an affront to their sense of autonomy.

 G11: If you forget an injection it can be useful when friends say, hey don't you have to do an injection? But it shouldn't be that someone asks about it every day, because it's my thing. (Girl, 14 years, social support type B)

*(2) Providing Help*. Some participants reported that their friends thought along with them or helped them out if something was wrong, for example, if they had a low blood glucose level or had forgotten their insulin pen. Several (particularly from the type A group) said that their friends' knowledge about diabetes was too limited to “really” be able to help, but some gave examples of help for which no specific knowledge of diabetes was required (e.g., letting the teacher know that the adolescent had left their insulin pen at home).

## 3. Study 2: Interviews with Adolescents with Diabetes and Their Best Friends

The first study showed that adolescents with diabetes primarily need emotional support from their friends but differ in the extent to which they are open to this form of social support. To gain more insight into the interpersonal contexts in which social support is provided, study 2 used in-depth interviews with adolescents with diabetes together with their best friends to examine the provision of social support. The main objective was to explore the experiences, expectations, and attitudes of adolescents with diabetes and their friends with respect to social support in coping with diabetes.

### 3.1. Methods

#### 3.1.1. Participants

Fifteen participants from study 1 were selected for participation in study 2 on the basis of gender and diversity of type of attitude towards support from friends (type A or B). Eleven of them (6 girls, 5 boys, aged 13–17) took part together with their best friend (7 girls, 4 boys, aged 13–19). With one exception, all pairs were of the same sex.  Table 2 shows background characteristics of the participants with diabetes.

#### 3.1.2. Data Collection

All interviews were conducted in the adolescents with diabetes' homes by the second author and tape-recorded. The interviews were semistructured and dealt with the following themes: friendship, impact of diabetes, diabetes, self-management, openness about diabetes and social support.

#### 3.1.3. Data Analysis

The interviews were transcribed verbatim and analysed with Kwalitan version 5.09 using content analysis [15–17]. The participants' perspectives were then grouped into the themes indicated above by a cyclical process of reflection, observation, and analysis. First 3 interviews were coded, on the basis of which the core themes were supplemented as needed with other important, recurring concepts. Then the remaining 8 interviews were coded and, finally, the coded text fragments were organized under the definitive themes.

### 3.2. Results

All pairs indicated that they were best friends or good friends. At the time of the interview 9 pairs attended the same school; the other 2 lived in the same neighbourhood. Most pairs saw each other daily, including outside school.

#### 3.2.1. Impact of Diabetes

Most of the adolescents with diabetes reported having little trouble with their diabetes, although tedious aspects were identified—particularly its ongoing character, injections, and hypo- and hyperglycaemia. Their friends recognized this but did not have the impression that the adolescents with diabetes were very limited by their diabetes.

 I: Do you ever get the impression that he's fed up with it?
 F6: No, because I don't think he is. Yeah, sometimes when it's not going so well, he gets tired of it. Especially with sports. I do think it's annoying for him that he has to stop because he has to do a test or something. (F6, boy, 14 years, friend of D6, boy with diabetes, social support type B)

The friends reported that the diabetes generally had little influence on their friendship. It was noteworthy that both the adolescents with diabetes and their friends were sensitive to word choice: words such as *burden* and *limited* were seen as signs of helplessness, with which the adolescents with diabetes did not want to be associated. Further, most of the adolescents with diabetes considered the word *support* to be an exaggerated description.

 I: What role does your friend play in coping with diabetes?
 D9: None. My friends don't support me and they also don't need to … He doesn't have to help me or anything… I'm not having a hard time, so then you don't need support. (D2, boy, 16 years, social support type B, and friend F2, 16 years)

#### 3.2.2. Experiences with and Need for Social Support

Different forms of emotional and instrumental support were mentioned in the interviews. As in study 1, more forms of emotional than instrumental support were reported. The adolescents with diabetes themselves did not generally refer to these forms as “support,” but rather saw them as “normal” aspects of friendship.

*Emotional Support*

*(1) Normal Treatment*. As discussed in study 1, for the adolescents with diabetes it was very important to be treated as normal. They were offended when people—other than their friends—assumed that they could not or may not do something or when they saw such people as interfering or as taking excessive account of their diabetes. Friends, too, felt that normal treatment was very important, and many recognized the irritation of the adolescents with diabetes.

 F7: Yeah, as a friend you just try to act normal. I mean you don't say “gosh, how awful”, but I mean you do show interest. Just don't overreact. (F7, friend of D7, girl with diabetes, social support type B, 16 years)

*(2) Distraction and Fun*. Most of the adolescents with diabetes indicated that “just having fun” with their friends was important to them; the friends confirmed this. Some girls, who said they did sometimes get fed up with the diabetes, went to their mothers rather than their friends in emotional situations and went to their friends when they were in need of distraction or cheering up. The adolescents with diabetes reported that their friends empathized with them and were considerate of their feelings, which they greatly appreciated. Some explicitly indicated that when with friends they wanted to have as little to do with their diabetes as possible.

*(3) Interest and Knowledge*. All of the adolescents with diabetes saw it in a positive light when friends showed interest in their diabetes, for example, by asking how it was going. Another sign of interest concerned friends having knowledge about diabetes-related matters, such as knowledge of the disease, knowledge of self-management tasks, awareness of the general welfare of the adolescent with diabetes, and recognizing when he did not feel well. Friends identified the same points under this theme.

*(4) Taking the Diabetes into Account*. For most of the friends, taking self-management tasks into account was simply a matter of course, for example, by waiting for their friend to administer an injection, planning meal times, or having diet products at home. Some friends occasionally found it irritating to have to keep such matters in mind but realized that it was necessary: in such situations they seemed to consider themselves as well as the adolescent with diabetes victims of the diabetes.

 F3: Sometimes I want to go with our friends somewhere, but they already head off, but I have to wait for [my friend with diabetes], that's annoying. And that she has to eat on time, that sometimes when we're shopping we can't go into the shop first, that's also annoying. (D3, girl, 13 years, social support type B, and friend F3, 13 years)

*Instrumental Support.* The comments concerning instrumental support involved providing help in case of emergencies and helping with and monitoring the execution of self-management tasks. None of the pairs had experienced an emergency together. All friends did have at least some awareness of what to do in case of emergency, and this was seen as important both by the adolescents with diabetes and by their friends. Further, adolescents with diabetes indicated that their friends had too little specific knowledge to be of great help with self-management tasks, such as counting carbohydrates or helping to give injections, which their friends confirmed. For their part, most of the friends did say that they paid attention to the adolescents with diabetes' self-management tasks and were sometimes worried when they knew things were not going well. Due to this concern several would have liked to be more involved, but this opportunity was in some cases withheld by the adolescents with diabetes as a result of their need for autonomy.

#### 3.2.3. Match between Need for and Provision of Support

For most pairs the interview appeared to be enlightening in the sense that it provided a better picture of the participants' wishes and needs. Most of the friends said they were willing to offer more support, should the adolescent with diabetes need it. In fact, some indicated that they would prefer this and said they did not offer more support only because the adolescent with diabetes did not appear to need it or was not overly open about the matter. In this sense, some friends wanted the adolescents with diabetes to be more open. The adolescents with diabetes themselves did not appear to need more support; they were afraid of coming across as nagging or needy or were worried about being a burden or felt the need to be autonomous. Their friends' willingness to provide more support suggests that these fears were largely unfounded.

## 4. Discussion

The results of the two studies described above share a certain amount of overlap and show that most of the adolescents with diabetes did not feel limited by their diabetes, despite the considerable impact it has on their lives. The friends' perceptions of the subjective impact of diabetes were generally similar to those of the adolescents with diabetes themselves. Both studies revealed various forms of emotional and instrumental support provided by friends that the adolescents with diabetes saw as supportive. Additionally, it was found that the adolescents with diabetes primarily need emotional support from friends, which supports earlier findings [11, 12, 20, 21].

An innovative finding is that the adolescents with diabetes differed in the involvement of their friends in their diabetes: some involved their friends very little (type A), whereas others did involve their friends to some degree (type B). This degree to which the adolescents with diabetes involved (and wanted to involve) their friends can help to explain why some adolescents with diabetes did not perceive some behaviours by friends as supportive whereas others did. Some forms of taking the diabetes into account, such as having diet products at home and showing solidarity (e.g., by not eating when their friend could not), were in fact seen as negative by some adolescents, as they gave them a sense of being treated differently or being a burden to others.

One of the major reasons of adolescents with diabetes for not being open or not wanting support was fear of the stigmas attached to illness and helplessness. Under no circumstances did they want to be seen as a patient; they were afraid of coming across as needy, different, or complaining and expressed the wish simply to be normal. These sentiments were stronger among those adolescents who did not involve or rarely involved their friends in their diabetes. Previous research has also identified this fear of stigmatization among adolescents with diabetes [18, 22].

The adolescents with diabetes' need for support was counterbalanced by their need for autonomy. As also found by Karlsson et al. [21], the adolescents saw their diabetes and self-management as their own business. This was reflected in the fine line that many of the adolescents with diabetes experienced between having their friends show interest on the one hand (e.g., occasionally asking about self-management tasks) and interfering on the other (e.g., being pushy). Some adolescents with diabetes, particularly those who did not involve or rarely involved their friends in their diabetes, showed a greater need for autonomy than others.

The relationship between attitudes towards support from friends and perceptions of social support warrants further investigation. Future research should expand the conceptualization of social support within peer relationships and consider interaction between person and contextual variables. For example, the strength or direction of the relationship between attitudes towards support from friends and perceptions of social support may depend on fear of stigmatization and sense of autonomy (see also [8]).

In addition, our data show that the views of the adolescents with diabetes and their friends were largely consistent, and in most pairs the need for and provision of support seemed to be in balance. The balance had developed over the course of the friendship, largely on the basis of implicit interactions, as support had rarely been discussed explicitly. Most friends were willing to offer more support, and the fear of adolescents with diabetes of asking for more support, motivated by the fear of stigmatization, seemed to be unfounded.

This research had various strengths and limitations. Strengths include the qualitative approach with its focus on the lived experience of adolescents, the mix of qualitative methods used, and the attention to interaction with friends. A limitation is that the research population was small and had relatively good blood glucose levels [23]. Adolescents who experience greater difficulty with their diabetes self-management may be less open about their diabetes and less likely to participate in research of this kind. Nevertheless, the study 1 participants had relatively diverse background characteristics, with the exception of ethnic background, and those in study 2 were selected from both social support types. However, caution is advised in generalizing the present results to all adolescents between the ages of 12 and 15 with type 1 diabetes.

Other limitations concern the data collection methods. A disadvantage of the online focus groups was that not all participants could take part on all days or at all times, which complicates claims about data saturation and the relationships between themes. In addition, online focus groups involve verbal communication only, and nonverbal signals may be missed. Nevertheless, online focus groups have important advantages compared to traditional focus groups: anonymous participation may promote greater openness [19], data can be processed faster and more accurately [24, 25], and participants can be brought together from geographically diverse locations. This latter point was crucial for us, given that discussions with the participating treating teams and adolescents with diabetes showed that it would be very difficult to recruit sufficient participants for traditional focus groups. Finally, it is possible that the participants in the pair interviews gave socially desirable answers and neglected to mention any potentially negative experiences. However, the simultaneous interviewing of adolescents with diabetes and their best friends gives a unique view on the interaction between the two.

## 5. Conclusions

The findings from our studies indicate that adolescents with diabetes receive various types of emotional support from friends. The adolescents differ as to the attitude towards friend involvement in their diabetes and as to which specific behaviours of friends they perceive as supportive or not. The adolescent's need for friend support often seems to match the actual support provided; friends are willing to give support. Sense of autonomy and fear of coming across as needy or different are major reasons for adolescents with diabetes for not being more open or not seeking more support.

To improve self-management and quality of life of adolescents with diabetes, diabetes education needs to pay attention to the social context in which self-management and disease adaptation take place, including relationships with friends. To this end, it is important that adolescents with diabetes learn the skills to decide for themselves how much support they want, particularly in view of their need for autonomy, and how to go about getting it. This could involve practical ways of informing others about their diabetes, being open about self-management tasks, and developing the skills to ask for support when needed. At present, some adolescents with diabetes may be less open with their friends than they may like to be due to fear of being a burden or of being stigmatized. The present results suggest that addressing this fear of stigmatization may help adolescents with diabetes to become more resilient. Friends can also be involved in interventions as a source of support, as was done earlier with some success [26].

## Figures and Tables

**Table 1:** Background characteristics of adolescents with diabetes in study 1.

Background data	Girls (*N* = 16)	Boys (*N* = 12)
Age mean (SD)	13.1 (1.0)	13.7 (1.7)
Ethnicity: native Dutch, nonmigrant	16 (100%)	12 (100%)

Educational level		
Secondary school (vocational level)	5 (31%)	1 (8%)
Secondary school (precollege level)	10 (63%)	5 (42%)
Postsecondary education (vocational)	—	1 (8%)
Not reported	1 (6%)	5 (42%)

Parental educational level		
Secondary school	7 (44%)	3 (25%)
Postsecondary education (vocational)	3 (19%)	2 (17%)
College/university	5 (31%)	2 (17%)
Not reported	1 (6%)	5 (42%)

Time since diagnosis		
<1 year	3 (19%)	2 (17%)
1–5 years	8 (50%)	3 (25%)
>5 years	5 (31%)	6 (50%)
Not reported	1 (6%)	1 (8%)

Insulin administration		
Pump	7 (44%)	3 (25%)
Injection	8 (50%)	8 (67%)
Not reported	1 (6%)	1 (8%)

Last HbA1c level		
<7.5 (optimal)	9 (56%)	4 (33%)
7.5–9.0 (suboptimal)	6 (38%)	1 (8%)
>9.0 (high risk)	—	2 (17%)
Not reported	1 (6%)	5 (42%)

**Table 2:** Background characteristics of adolescents with diabetes in study 2.

Background data	Girls (*N* = 6)	Boys (*N* = 5)
Age mean (SD)	14.3 (1.2)	15.4 (1.3)
Ethnicity: native Dutch, nonmigrant	6 (100%)	5 (100%)

Educational level		
Secondary school (vocational level)	2 (33%)	—
Secondary school (precollege level)	4 (67%)	3 (60%)
Postsecondary education (vocational)	—	1 (20%)
Not reported	—	1 (20%)

Parental educational level		
Secondary school	1 (17%)	2 (40%)
Postsecondary education (vocational)	1 (17%)	1 (20%)
College/university	4 (67%)	1 (20%)
Not reported	—	1 (20%)

Time since diagnosis		
1–5 years	2 (33%)	2 (40%)
5–10 years	2 (33%)	2 (40%)
>10 years	2 (33%)	1 (20%)

Insulin administration		
Pump	1 (17%)	2 (40%)
Injection	5 (83%)	3 (60%)

Last HbA1c level		
<7.5	2 (33%)	3 (60%)
7.5–9.0	3 (50%)	1 (20%)
>9.0	—	1 (20%)
Not reported	1 (17%)	—

Social support attitude		
Type A (low friend involvement)	2 (33%)	3 (60%)
Type B	4 (67%)	2 (40%)

Friendship duration		
1-2 years	1 (17%)	—
2–5 years	2 (33%)	2 (40%)
5–10 years	2 (33%)	1 (20%)
>10 years	1 (17%)	2 (40%)
